# Low-Viscosity Polydimethylsiloxane Resin for Facile 3D Printing of Elastomeric Microfluidics

**DOI:** 10.3390/mi14040773

**Published:** 2023-03-30

**Authors:** Elyse Fleck, Charlise Keck, Karolina Ryszka, Emma DeNatale, Joseph Potkay

**Affiliations:** 1 ECLS Laboratory, Department of Surgery, University of Michigan, Ann Arbor, MI 48109, USA; 2 VA Ann Arbor Healthcare System, Ann Arbor, MI 48105, USA

**Keywords:** 3D printing, digital light processing, microfluidics, microfabrication, Poly(dimethylsiloxane)

## Abstract

Microfluidics is a rapidly advancing technology with expansive applications but has been restricted by slow, laborious fabrication techniques for polydimethylsiloxane (PDMS)-based devices. Currently, 3D printing promises to address this challenge with high-resolution commercial systems but is limited by a lack of material advances in generating high-fidelity parts with micron-scale features. To overcome this limitation, a low-viscosity, photopolymerizable PDMS resin was formulated with a methacrylate-PDMS copolymer, methacrylate-PDMS telechelic polymer, photoabsorber, Sudan I, photosensitizer, 2-isopropylthioxanthone, and a photoinitiator, 2,4,6-trimethyl benzoyl diphenylphosphine oxide. The performance of this resin was validated on a digital light processing (DLP) 3D printer, an Asiga MAX X27 UV. Resin resolution, part fidelity, mechanical properties, gas permeability, optical transparency, and biocompatibility were investigated. This resin produced resolved, unobstructed channels as small as 38.4 (±5.0) µm tall and membranes as thin as 30.9 (±0.5) µm. The printed material had an elongation at break of 58.6% ± 18.8%, Young’s modulus of 0.30 ± 0.04 MPa, and was highly permeable to O_2_ (596 Barrers) and CO_2_ (3071 Barrers). Following the ethanol extraction of the unreacted components, this material demonstrated optical clarity and transparency (>80% transmission) and viability as a substrate for in vitro tissue culture. This paper presents a high-resolution, PDMS 3D-printing resin for the facile fabrication of microfluidic and biomedical devices.

## 1. Introduction

The miniaturization of technology, most notably electronic microsystems, has paved the way for the expansion of microfluidics as a new class of devices across all industries. For decades, microfluidics has been explored as a means to improve portability and analytical sensitivity while reducing time, cost, reagents, and energy consumption [1,2]. While their applications are expansive, from gas phase chromatography and digital polymerase chain reaction (dPCR) to organs-on-a-chip, the fabrication of microfluidics is still laborious, thus restricting their wider use [2,3,4,5]. The common microfabrication techniques include soft lithography, micromilling, and glass etching, but these methods require many steps with highly skilled labor, and device designs are restricted to planar geometries, preventing higher-throughput systems from being developed [1,6,7,8].

Currently, 3D printing promises to address many of these challenges due to its user-friendly automation and 3D design capabilities, which overcome the planar geometry constraints of traditional approaches [9,10,11,12]. Of the many available 3D printing technologies, vat photopolymerization via stereolithography (SLA) and digital light processing (DLP) have become popular choices for making microfluidic devices due to their micron-scale resolution, practical build volume, and open material systems [13,14]. This has motivated the development of custom resins capable of producing highly resolved features, specifically channels and membranes, to produce microfluidic devices that are truly on the micron scale [13,15,16,17,18,19].

Despite many advancements, there is a lack of high-resolution polydimethylsiloxane (PDMS)-based resin suitable for DLP. PDMS, namely Sylgard 184, has become a ubiquitous material in microfluidics due to its functionality in soft lithography methods [1,20] and many attractive properties including chemical inertness, high gas permeability, low polarity, low electrical conductivity, elasticity, optical clarity, and transparency in the ultraviolet and visible regions [21]. While Sylgard 184 offers many advantages, it is not a suitable choice for large-scale manufacturing processes such as injection molding to create microfluidic devices, restricting its value in the field. The creation of a photopolymerizable PDMS for microfabrication via DLP would be a significant contribution to the field of microfluidics to mitigate the manufacturing drawbacks of soft lithography and increase device throughput.

Various groups have made progress in developing PDMS-based resins for DLP, but the smallest membranes reported in literature are >330 μm [22], and the smallest channels are 400 μm [23]. Our laboratory previously developed a novel PDMS resin that exhibits many of the advantageous properties of Sylgard 184 including optical transparency and gas permeability while achieving highly resolved micron-scale parts (60 μm channels and 20 μm membranes) [19]. However, that resin exhibited printability challenges due to its high viscosity and brittle parts.

Resin printability via surface-constrained DLP, also known as bottom-up projection, and the approach used in this study are especially dependent on the viscosity of the material, where large separation forces pulling against the part during printing can easily lead to part defects or build failures [24]. The advantages of surface- constrained DLP over free-surface DLP (also known as top-down projection) are thinner layer thicknesses and small resin vat volumes, both of which are desirable for the fabrication of microfluidic devices [12,24]. Reducing the overall viscosity of the resin by the addition of a photopolymerizable diluent is one approach to mitigate the potential challenges caused by surface-constrained separation forces. This paper presents an improved resin with low viscosity and elastomeric parts, enhancing its printability while maintaining the high fidelity of micron-scale features, permitting the facile 3D printing of microfluidic devices.

## 2. Results

### 2.1. Resin Formulation and Print Characterization

**Figure 1:**
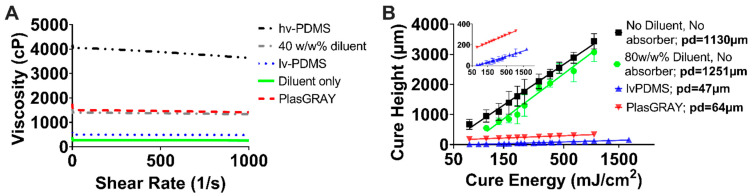
(**A**) Viscosity curves (*n* = 3 for each curve) demonstrating the decrease in viscosity with the addition of diluent DMS-R22. All curves contain TPO-L, Sudan, ITX, DMS-R22, and RMS-083. Exact w/w% values are listed in Table S1. PlasGRAY is a high-resolution resin sold by Asiga (Asiga, Alexandria, NSW 2015) and included as a reference point. (**B**) Working curves of cure energy vs. cure height to determine the characteristic penetration depth of the resin, *p_d_* = 47 µm (*n* ≥ 9).

A methacryloxypropyl-terminated PDMS telechelic polymer, DMS-R22, was incorporated into our previous, high-viscosity resin formula to function as a diluent. Because greater than 5 mole % of methacrylate substitution is required for UV-induced crosslinking of a nonflowable resin [25], the weight fraction (w/w%) of DMS-R22 and PDMS copolymer, RMS-083, was considered when determining the lowest feasible viscosity of the resulting polymer mixture. Rheology was performed on various w/w% combinations of RMS-083 and DMS-R22 to determine the lowest viscosity that could still achieve photopolymerization. Given these considerations and the viscosity curves presented in Figure 1A, 80 w/w% DMS-R22 was selected as the optimum fraction of diluent for further characterization.

Various w/w% combinations of Sudan I in the 80 w/w% DMS-R22 resin formula were tested to increase resolution while maintaining a stable resin formulation. Above 0.1 w/w% of Sudan I and 0.4 w/w% of 2-isopropylthioxanthone (ITX), large aggregates and precipitates (>100 µm) began to form in the resin, making it unsuitable for practical use as a 3D printing resin. A formula of 0.09 w/w% Sudan I, 0.40 w/w% ITX, 0.80 w/w% 2, 4, 6-trimethyl benzoyl diphenylphosphine oxide (TPO-L), 18.71 w/w% RMS-083, and 80.00 w/w% DMS-R22 demonstrated stability and is referred to as low-viscosity PDMS (lv-PDMS). For comparison, the previous high-viscosity version of this resin (without DMS-R22) [19], was prepared with 0.20 w/w% Sudan I, 0.40 w/w% ITX, 0.80 w/w% TPO-L, and 98.60 *w*/*w*% RMS-083 and is referred to as high-viscosity PDMS (hv-PDMS).

A working curve to determine the characteristic penetration depth, *p_d_*, of the material is given in Figure 1B, where *h_c_* is the cure height or thickness of polymerized resin, *E_c_* is the cure energy exposed from the light source, and *p_d_* is calculated from the slope of the curve given the relationship in Equation (1) [19,22,26,27].

(1) $$h_{c}=p_{d}ln\;\left(E_{c}\right)\;$$

This relationship is a widely accepted predictor of 3D printing resin resolution, where *p_d_* describes the depth that light can penetrate the resin before being absorbed by the material, allowing polymerization to occur. This effect can beneficially attenuate bleeding light into adjacent features or previous layers during printing, avoiding the undesired curing of resin trapped in regions such as microfluidic channels. From the plot in Figure 1B, the resolution of this resin, lv-PDMS, is *p_d_* = 47 µm. This was compared with a commercially available, high-resolution (not gas-permeable) resin, PlasGRAY, which has a penetration depth of 64 µm. To create truly micron-scale features, a smaller *p_d_* enables more precise control of the photopolymerization reaction. When fabricating microfluidic devices, even slight variations in the *p_d_* (1–5 µm) can be impactful. A smaller *p_d_* enables tighter tolerances and therefore highly resolved internal features in the microfluidic device.

### 2.2. Print Resolution

**Figure 2:**
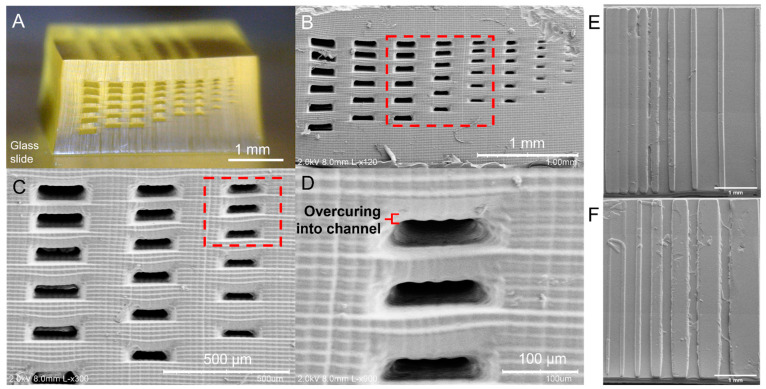
(**A**) Microfluidic characterization print showing cleared channels. (**B**–**D**) Front-view SEM images of the highly resolved micron-scale channels. Red box in (**B**) highlights selected channels dimensioned for resolution performance. Summary available in Figure S2. Red box in (**C**) identifies zoomed-in region shown in (**D**). (**D**) Overcuring into the channel is evident and highlighted on the image with a red bracket. (**E**,**F**) XY pixel resolution parts with (**E**) constant pillar width and (**F**) constant spacing. Summary of XY resolution is provided in Figure S2.

To further characterize the print resolution of lv-PDMS, a part with an array of micron-scale, rectangular channels ranging from 40 to 140 µm tall and membranes ranging from 20 to 100 µm thick was printed on an Asiga MAX X27 UV DLP printer (Figure 2). The microfluidic characterization print produced clear channels as small as 38.4 µm tall (±5.0 µm) and membranes as thin as 30.9 µm (±0.5 µm). Channels that were successfully cleared of uncured resin are shown in Figure 2A, where the lighter striations along the top of the part indicate the patent, resin-free channels. SEM images were taken to verify the designed channel dimensions vs. the actual printed dimensions and to determine the print resolution (red dashed box in Figure 2B; results in Figure S2). Figure 2D depicts a zoomed-in view of the channels highlighted by the red dashed box in Figure 2C, clearly showing the layer-by-layer fabrication from the horizontal striations across the face of the part. Also notable in Figure 2D is the overcuring of the resin into the channel because of light bleeding from the subsequently printed layers. XY resolution was also characterized with two versions of XY pixel resolution prints designed with 5 mm long pillars of constant width and varying spacing (Figure 2E) and pillars of varying widths with constant spacing (Figure 2F). A detailed summary of the results is presented in Figure S2. Features spaced closer than 10 pixels (270 µm) apart and features less than 5 pixels (135 µm) wide were not resolved.

### 2.3. Mechanical Properties

**Figure 3:**
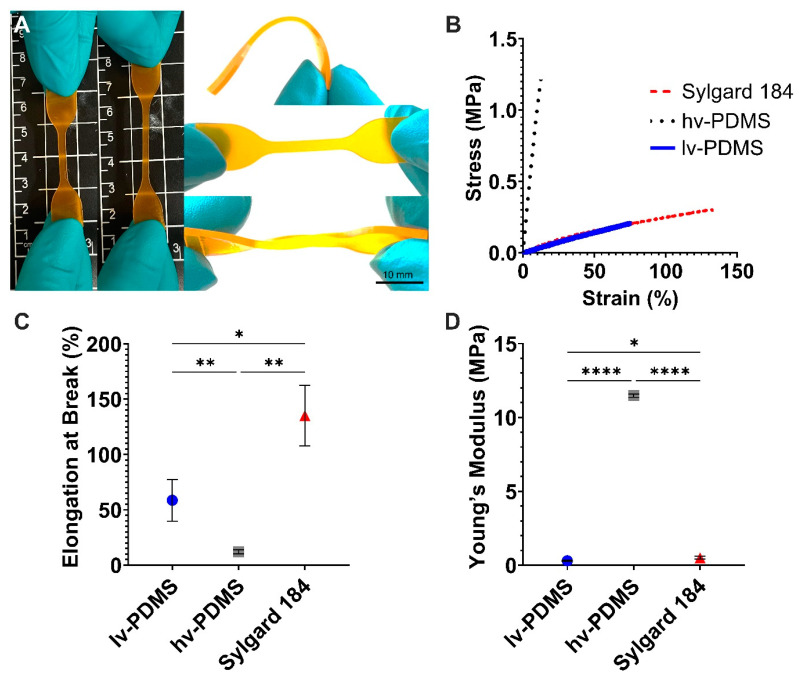
(**A**) A 3D-printed tensile bar with lv-PDMS resin demonstrating elastic behavior. (**B**) Stress–strain curves from tensile testing of Sylgard 184 (red, dashed line), hv-PDMS (black, dotted line), and lv-PDMS (blue, solid line) (*n* = 5). (**C**) Calculated elongation at break (*n* = 5; * indicates *p* < 0.05, and ** indicates *p* < 0.01). (**D**) Calculated Young’s modulus (*n* = 5; * indicates *p* < 0.05, and **** indicates *p* < 0.0001).

The addition of diluent, DMS-R22, to the resin formula also improved the 3D-printed material’s mechanical properties. Our previous hv-PDMS resulted in a brittle and rigid polymer. The lv-PDMS formula is more elastic, approaching the mechanical properties of Sylgard 184, with the exception of elongation at break, as shown in Figure 3. Despite a smaller elongation, the lv-PDMS still maintains elastomeric behavior, as shown in Figure 3A and Supplementary Video S1 of the tensile bar being stretched and bent without tearing. The exact mechanical properties are listed in Table 1.

### 2.4. Gas Permeability

**Figure 4:**
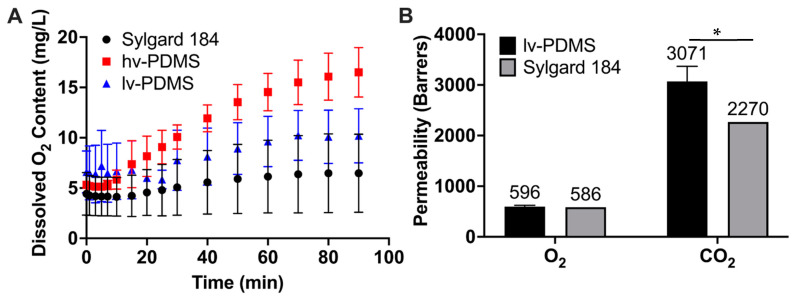
(**A**) Dissolved O_2_ measurements demonstrating gas permeability of the cured resins, lv-PDMS and hv-PDMS, relative to Sylgard 184. Error bars represent standard deviation (*n* = 3). A dose–response regression of the curves showed that the total oxygen content (top plateau) between all groups was statistically significant (*p* < 0.0001). (**B**) Gas permeability measurements taken at Labthink International Inc. of 150 µm film made from lv-PDMS resin. Error bars represent standard deviation (*n* = 3). O_2_ standard deviation is 28 Barrers, and CO_2_ standard deviation is 295 Barrers. Sylgard 184 data were taken from the literature [28]. A one-sample t-test comparing lv-PDMS with Sylgard 184 determined there was not a significant difference for O_2_ but there was for CO_2_ (* indicates *p* < 0.05).

The lv-PDMS resin also maintained high gas permeability to O_2_ and CO_2_, as was previously demonstrated with the hv-PDMS resin [19]. Using an established method [19], O_2_ permeability was measured by applying a fixed pressure of O_2_ to a 100 µm membrane made of cured resin and measuring the dissolved O_2_ content over time in a fixed reservoir of deionized water on the other side of the membrane. As shown in Figure 4, lv-PDMS resin had a smaller O_2_ content over time than the hv-PDMS resin. However, it performed as well as Sylgard 184, demonstrated by the higher levels of O_2_ content across all timepoints.

To further validate these results, 150 µm lv-PDMS membranes were tested at a third-party company, Labthink International Inc., to measure the gas permeability of CO_2_ and O_2_ according to ASTM D1434-82(R09)^e1^. The results given in Figure 4B demonstrate that lv-PDMS has gas exchange comparable to that of the commercial standard, Sylgard 184, as predicted by the dissolved O_2_ measurements.

### 2.5. Postprocessing via Solvent Extraction

**Figure 5:**
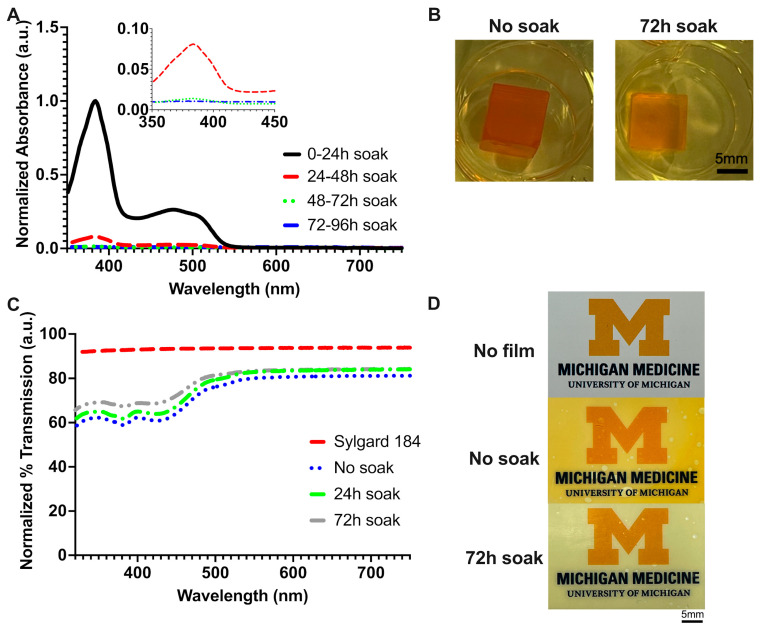
(**A**) Absorbance signal of extraction media after soaking 3D-printed part in EtOH (*n* = 3), (**B**) comparison of the color loss from a cube that was extracted with EtOH (72 h soak) to one that was not extracted (no soaking), (**C**) transmission data of 100 µm thin films after extraction with EtOH (*n* = 3), and (**D**) optical clarity of films after 72 h of EtOH extraction relative to no soak (no EtOH extraction) and no film (original image of Michigan Medicine logo) treatments.

The extraction of unreacted monomers and photoabsorbing compounds is a critical postprocessing step to ensure the visual clarity and transmission of the cured polymer as well as possible biological applications where toxicity and/or biocompatibility is concerned. In this study, 3D-printed 1 cm^3^ cubes with lv-PDMS resin were soaked in ethanol (EtOH) for a total of 96 h to determine the adequate length of time in solvent to extract these compounds. Figure 5A shows a plot of absorbance curves decreasing over time, where, after 72 h, the absorbance signal of the extractable compounds in the EtOH solution substantially reduced, indicating the removal of these unwanted constituents. The removal of these constituents is visibly apparent in Figure 5B, which compares a part that was soaked in EtOH for 72 h with one that was not.

The transmission of 100 µm 3D-printed films of lv-PDMS was also measured before and after soaking in EtOH for 24 h and 72 h to demonstrate the optical clarity of the resin after the extraction of the unreacted monomers, photoinitiator, and photoabsorbing compounds. Figure 5C shows that solvent extraction with EtOH enabled 80% transmission in the visible light spectrum relative to Sylgard 184. Optical clarity of lv-PDMS is demonstrated in Figure 5D comparing the 72 h EtOH extracted film overlaid on a Michigan Medicine logo with a film that was not extracted with EtOH (no soak) and the original image (no film).

### 2.6. Biocompatibility and Microfluidic Applications

**Figure 6:**
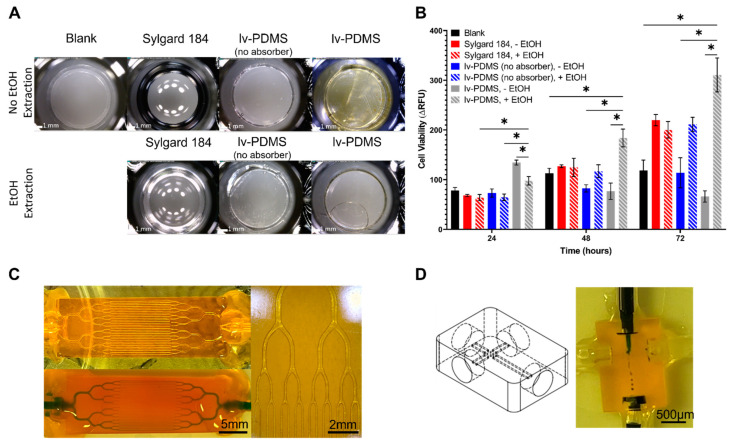
(**A**) Top-down view of a 96-well plate with polymer discs at the base of the well (no cells). Samples had no EtOH extraction and a 72 h EtOH extraction. (**B**) Cell viability of HepG2 cells grown on blank wells, Sylgard 184, lv-PDMS (no absorber), and lv-PDMS. All polymer samples were extracted with ethanol (+EtOH) or not extracted (-EtOH) (*n* = 3). A 2-way ANOVA with Tukey’s multiple comparison test determined a significant interaction (*p* < 0.0001) between the effects of treatment (control, untreated, and extracted) and time (24 h, 48 h, and 72 h) on cell viability (ΔRFU). Tukey’s multiple comparisons for all sample combinations can be found in Table S3. Only comparisons with (* indicates *p* < 0.05) for the EtOH-extracted lv-PDMS resin are denoted on the plot for sake of brevity. (**C**) Biomimetic branching network of 200 µm tall capillaries (top) and device with blue food dye flowed through (bottom). Zoomed-in image of microchannels void of resin (right). (**D**) Droplet generator with 200 µm tall channels. CAD rendering (left) showing internal channels and droplets were composed of blue-food-dyed water in oil (right).

The possibility of lv-PDMS resin being used for biological applications such as cell culture was studied by 3D-printing disc inserts that would sit at the bottom of the wells in a 96-well plate. A solvent extraction was carried out with EtOH for 72 h to remove potentially toxic compounds in the material (Figure 6A). Cell viability studies were performed with HepG2 cells grown on blank wells, Sylgard-184-coated wells with and without an EtOH extraction, lv-PDMS (no photoabsorbers) with and without an EtOH extraction, and lv-PDMS with and without an EtOH extraction. The results in Figure 6B show that the removal of unreacted groups and photoabsorbing compounds (lv-PDMS, +EtOH) had a significant effect (*p* < 0.05), improving cell growth and viability after 48 h and 72 h relative to the blank wells and the no EtOH extraction groups (lv-PDMS, -EtOH and lv-PDMS (no absorber), -EtOH).

Various microfluidic devices were printed to demonstrate the wide application of this lv-PDMS. Figure 6C shows a complex, biomimetic capillary branching structure with 200 µm tall channels. Figure 6D depicts a droplet generator with 200 µm tall channels creating water-in-oil-droplets. This resin enables the facile assembly of connections to the microfluidic devices by directly printing holes or connectors where the tubing can be easily sealed.

## 3. Discussion

Our previous hv-PDMS resin was able to print highly resolved channels and membranes but had a large viscosity [19], which led to printing challenges from separation forces of the viscous resin pulling at the part during a build [24]. This challenge often resulted in tearing of the part and print failures. The printability of this resin was improved by the addition of a photopolymerizable diluent to the formulation, resulting in a high-resolution and low-viscosity resin, lv-PDMS. A lower-viscosity resin also enables easier clearing of uncured resin from the microchannels during postprocessing of the printed parts.

The penetration depth of lv-PDMS, *p_d_* = 47 µm, enabled 3D printing of the highest resolved channels (38.4 µm tall ± 5.0 µm) and membranes (30.9 µm ± 0.5 µm) with a PDMS-based resin to date, excluding our previous hv-PDMS, which resulted in similar resolved dimensions. This achievement is one-tenth the size of the previously reported 400 µm channels [23] and 330 µm membranes [22] printed with PDMS resins by other research groups. Table 2 highlights this achievement with respect to other high-resolution microfluidic 3D printing resins for DLP.

Further illustrated in Table 2, lv-PDMS has many of the advantageous properties of Sylgard 184 that are utilized in microfluidics. Printing with the previous hv-PDMS resulted in a rigid and brittle material due to its low elongation at break and high Young’s modulus, limiting its usefulness in microfluidic applications. The enhanced elasticity of lv-PDMS compared with that of the previous hv-PDMS would allow the creation of pneumatic valves to control fluid flow in microfluidic devices. The excellent gas permeability of lv-PDMS was maintained from the previous version of the formula and is a suitable replacement for Sylgard 184, unlike the poly(ethylene glycol) diacrylate (PEGDA) resins presented in Table 2. While PEGDA’s high-resolution printability is attractive, its gas permeability is an order of magnitude smaller than that of PDMS for O_2_ and two orders of magnitude smaller for CO_2_ [29]. Given this, PEGDA has not often been investigated or utilized for its gas permeability and may not be a suitable alternative to Sylgard 184 depending on the application. Optical clarity and transparency (>80% transmission relative to Sylgard 184) in the visible-light spectrum permits the visibility of internal structures in the 3D-printed device for microscopy purposes or signal detection.

Sylgard 184 is also a choice material for its biocompatibility with in vitro tissue culture [30]. Microfluidics has become increasingly attractive in biological applications to study and model cellular microenvironments with precision [31]. The extraction of unreacted molecules and toxic photoabsorbing compounds from 3D-printed resins is known to improve the biocompatibility of 3D-printed parts [32], but until the creation of lv-PDMS, no PDMS-based resins had high enough resolution to create microfluidic devices with features at truly micron-size scales [22]. The solvent extraction with ethanol of parts 3D-printed with lv-PDMS demonstrated high cell viability in HepG2 cells. The no-ethanol-extraction inserts had the lowest cell viability due to the toxic compounds still present in the cured polymer, warranting further investigation into the biocompatibility of this new material. Additional testing should be performed with other cell types to further validate this material’s cytocompatibility.

With the promise of a biocompatible resin, we anticipate that cell-based microfluidics can be created using lv-PDMS. The microfluidic network in Figure 6C is a demonstration of the ability to mimic the branching and vessel scaling of the natural vasculature. The introduction of cells to this gas-permeable scaffold offers many potential applications from modeling microvasculature to organ-on-a-chip devices. The functionality of this resin to create devices such as the droplet generator presented in Figure 6D highlights the versatility of lv-PDMS for the field of microfluidics.

## 4. Materials and Methods

### 4.1. Resin Formulation

Resin components were weighed on a Quintix 125D-1S Semi-Micro Balance (Sartorius Lab Instruments GmbH & Co. KG, Goettingen, Germany) to the selected w/w% of material. Components were combined and mixed by hand, heated for 2 h at 50 °C on a VMS-C7 S1 hot plate (VWR International, Radnor, PA, USA), and then sonicated with a Q700 sonicator (Qsonica LLC, Newtown, CT, USA) to ensure uniform mixing and particle size reduction. Sudan I (photoabsorber) was purchased from Sigma-Aldrich (St. Louis, MO, USA). 2-Isopropylthioxanthone (ITX; photosensitizer) ≥ 98.0% was purchased from VWR International (Radnor, PA, USA). We purchased 7–9% (methacryloxypropyl)methylsiloxane]-dimethylsiloxane copolymer (RMS-083; side-chain polymer) and methacryloxypropyl terminated polydimethylsiloxane, 125–250 cSt (DMS-R22; end-chain polymer) from Gelest, Inc. (Morrisville, PA, USA). 2,4,6-Trimethyl benzoyl diphenylphosphine oxide (TPO-L; photoinitiator) was gifted as a sample from PL Industries of Esstech, Inc. (Essington, PA, USA).

To determine the maximum concentration of DMS-R22 that would permit polymerization while minimizing resin viscosity, 0.8 w/w% TPO-L was combined with various w/w% combinations of DMS-R22 (10, 20, 40, 80, 90, and 99.2 w/w%) and RMS-083 (89.2, 79.2, 59.2, 19.2, 9.2, and 0 w/w%). We created 5 g batches with the appropriate amounts of TPO-L, DMS-R22, and RMS-083 as described above, which we exposed to 385 nm light at 15 mW/cm^2^ for 30 s in an Asiga MAX X27 UV DLP 3D printer (Asiga, Alexandria, Australia) purchased from Proto Products (Ashland City, TN, USA). The resin mixture was sandwiched between two glass slides during exposure to prevent oxygen inhibition from free radical scavenging. We determined 80 w/w% R22 as the maximum amount that permitted polymerization. To maximize resolution, Sudan I and ITX concentrations were increased in the resin until their solubility limits were reached (determined by the formation of visible precipitates). A resin consisting of 80.0 w/w% DMS-R22, 18.71 w/w% RMS-083, 0.80 w/w% TPO-L, 0.40 w/w% ITX, and 0.09 w/w% Sudan I was formulated for further testing as described next.

### 4.2. Rheology

The viscosity of resins with varying w/w% diluent was determined using a TA Discovery HR-2 Rheometer (TA Instruments, New Castle, DE, USA) at the University of Michigan’s Battery Lab, Ann Arbor, MI, USA. A flow sweep of the samples was run using a 20 mm parallel Peltier steel plate and a 1 mm gap. The weight fractions (w/w%) of the samples are listed in Table S1. The rheology data (*n* = 3) for each sample group were averaged and plotted with GraphPad Prism software version 9.1.0 (GraphPad Software, San Diego, CA, USA).

### 4.3. Resin Curing Dynamics

The resin curing dynamics were determined by placing uncured resin on a glass slide and cured by exposing a small circle of light (5.2 mm^2^) from the printer (MAX X27 UV) at 15 mW/m^2^ at various time points. The excess uncured resin was rinsed from the glass slide with ACS-grade ≥99.5% isopropyl alcohol (IPA) (LabChem, Zelienople, PA, USA) purchased from Fisher Scientific Company (Hampton, New Hampshire, USA). The thickness of the cured resin was measured by taking side-view images of the cured spot with an AM413T Dino-Lite Digital Microscope using DinoCapture 2.0 software (Dunwell Tech, Inc., Torrance, CA, USA; camera resolution was ±3 μm). Resins were cured and measured at least three separate times with triplicate measurements taken for each thickness (*n* ≥ 9). Simple linear regressions were run in GraphPad Prism to determine the slope, x-intercepts, and standard error of these curves. Cure energy and resulting thickness were input into a material file for printing with an Asiga MAX X27 UV printer.

### 4.4. Printing Resolution Characterization

All resin characterization and parts were printed using an Asiga MAX X27 UV printer. This printer used DLP technology with a 385 nm LED light source (wavelength range of 370–400 nm), an X and Y pixel resolution of 27 μm, and a Z (vertical) resolution of 1 μm. Asiga Composer Software version 1.2.12 (Asiga, Alexandria, NSW 2015) was used as the interface for handling STL files and controlling print parameters. We generated 3D models in SOLIDWORKS (Dassault Systems, Waltham, MA, USA) and exported to STL file format. A microfluidic characterization print consisting of an array of channels with varying heights (40–140 μm) separated by membranes with varying thicknesses (20–100 μm) was designed to test the ability of the resin to achieve microfluidic features of various sizes; see Figure S1. Two versions of an XY pixel resolution print were designed with varying spacing between pillars (27–81 μm) with constant pillar width (162 μm; version 1) and varying pillar width (81–810 μm) with constant spacing (270 μm; version 2); see Figure S2. Glass slides were silanized with 3-(trimethoxysilyl)propyl methacrylate (Sigma Aldrich, St. Louis, MO, USA) following the procedure as described by Urrios et al. [33], then attached to the build platform using UV epoxy (Proto Glass, Proto Products, Ashland City, TN, USA) at the start of each print to ensure adhesion of the build to the build platform while creating a smooth build surface. Contact angle measurements using deionized water were used to verify the silanization of the slides, as previously described [34]. The build platform was leveled to the printer with the glass slide attached and then printing proceeded as normal.

Successful prints were recovered from the build platform by removing the glass slide, which were then soaked in IPA to wash away most of the uncured resin. For the microfluidic characterization prints, a vacuum was applied to the open end of the channels to suction out the residual uncured, liquid resin. After uncured resin was removed, the part was post cured in an Asiga Flash-type DR-301C UV exposure chamber (Asiga, Alexandria, NSW 2015) purchased from Proto Products (Ashland City, TN, USA). The microfluidic characterization print and XY pixel resolution prints were measured using a Hitachi SU8000 In-line FE-SEM at the Lurie Nanofabrication Facility at the University of Michigan (Ann Arbor, MI, USA). Image J Software (U. S. National Institutes of Health, Bethesda, Maryland, USA) version 1.51 [35] was used to collect the dimensions of the channels for the quantification of the print resolution from the SEM images (Figure S2). Descriptive statistics of mean, standard deviation, and standard error of mean were calculated in Excel (Microsoft, Redmond, Washington, USA) (*n* = 6 for microfluidic characterization print measurements and *n* = 3 for XY pixel resolution prints).

### 4.5. Mechanical Testing

Mechanical testing was performed via tensile testing using a TA.XTPlus Texture Analyser and Exponent Connect software version 6 (Texture Technologies, Hamilton, MA, USA) at the Van Vlack Laboratory at the University of Michigan. The testing setup and specifications are detailed in Figure S3 and Video S2. Tensile bars were constructed according to ASTM D412 but scaled to fit the build area of the Asiga printer (Figure S4). Tensile bars were directly printed onto the build platform, then removed and washed in IPA before postcuring in an Asiga Flash-type DR-301C UV exposure chamber. Dow Sylgard 184 (purchased from Ellsworth Adhesives, Germantown, WI, USA) tensile bars were formed by filling an acrylic mold (Figure S4) with a degassed 10:1 polymer to crosslinker mixture and baked for 1 h at 80 °C. The acrylic mold was formed via laser etching a 3.175 mm thick acrylic sheet (Professional Plastics, Inc., Fullerton, CA, USA) using a Zing 16 laser engraver (Epilog Laser, Golden, CO, USA) and CorelDRAW 2017 software version 19 (Corel Corporation, Ottawa, ON, Canada). All tests were performed with *n* = 5. Descriptive statistics and one-way ANOVA (α < 0.05) with post hoc Tukey tests were calculated using GraphPad Prism. Plots were generated in GraphPad Prism.

### 4.6. Gas Permeability

Gas permeability was tested using a previously established method, and the setup for this system can be found in Figure S3 of Fleck et al. [19]. Briefly, oxygen (O_2_) permeability was qualitatively measured by applying 2 psi of fixed pressure of O_2_ to one side of a thin 3D-printed film while measuring dissolved O_2_ concentration for 90 min via a Milwaukee MW600 PRO Dissolved Oxygen Meter (Milwaukee Instruments Inc., Rocky Mount, NC, USA) in a fixed volume of DI water on the other side of the film. The testing setup can be found in Fleck et al.’s Supplementary Figures [19]. PDMS resin-based films were 100 μm thick printed on an Asiga MAX X27 UV printer. Sylgard 184 films were 100 μm thick and formed using an SCS G3P-12 Spin Coater (Specialty Coating Systems Inc., Indianapolis, IN, USA) with a 10:1 polymer to crosslinker mixture and baked for 1 h at 80 °C. Gas permeability plots were generated in GraphPad Prism (*n* = 3 for all test groups), and error bars represent the standard deviation. A dose–response regression for variable slopes was performed in GraphPad Prism to compare the top plateau of the curve across groups. This was chosen to compare the total dissolved oxygen content.

Gas permeability was quantitatively measured by Labthink International, Inc. (Labthink Instruments Co., Ltd., Jinan, China) for O_2_ and CO_2_ gas permeability according to ASTM D1434-82(R09)^e1^. Films were prepared by exposing a build tray of resin to UV light from the entire build area of an Anycubic Mono X printer (Shenzhen, Guangdong, China) at 2.5 mW for 200 s to make 192 × 120 mm films, 150 µm thick. Films were washed in IPA and postcured in an Asiga Flash-type DR-301C UV exposure chamber for 5 min on each side. Samples were then shipped to Labthink International Inc. for testing. Data were plotted in GraphPad Prism (*n* = 3 for test groups), and error bars represent standard deviation. A one-sample t-test was performed to compare lv-PDMS with Sylgard 184 (α < 0.05).

### 4.7. Ethanol Extraction of Unreacted Resin Components from Printed Parts

We printed 1 cm^3^ cubes on an Asiga MAX X27 UV without a silanized glass slide (directly onto the build platform) to facilitate easier removal of the part. Cubes were soaked for 96 h in anhydrous ethanol (Fisher Scientific Company, Fair Lawn, NJ, USA) at room temperature. At each 24 h time point, the supernatant was collected and replaced with fresh ethanol. Samples (*n* = 3) were diluted 1:1000 in ethanol before spectrophotometry.

Absorbance measurements of extraction samples were taken from 350 to 750 nm using a Varian Cary 50 Bio UV-Vis spectrophotometer (Agilent Technologies, Santa Clara, CA, USA). Triplicates of each sample were run in Hellma^®^ absorption cuvettes (Hellma GmbH & Co., Müllheim, Germany) made of Herasil quartz with a spectral range of 260–2500 nm, pathlength of 10 mm, and chamber volume 3500 μL purchased from Sigma Aldrich (St. Louis, MO, USA). Absorbance data (*n* = 3) for each sample group were averaged, and then the dataset was normalized to a range of (0, 1). Plots were generated in GraphPad Prism.

### 4.8. Optical Transmission

Optical transmission measurements were taken from 350 to 750 nm using a Varian Cary 50 Bio UV-Vis spectrophotometer. We printed 100 μm films of each resin formulation on an Asiga MAX X27 UV printer on uncoated glass slides to reduce surface roughness and ensure film transparency. All printed films were rinsed with IPA to remove excess resin. Films for the no-soak group were set aside, and ethanol soaked films were soaked for 24 h or 72 h, exchanging fresh ethanol every 24 h. Sylgard 184 films were formed using an SCS G3P-12 Spin Coater, with a degassed 10:1 polymer to crosslinker mixture and baked for 1 h at 80 °C. Measurements were taken by securing the thin film onto the cuvette holder in the spectrophotometer so that the film intercepted the laser source for detection (no cuvette was used). The transmission data for each sample group (*n* = 3 films) were averaged, and then the dataset was normalized to a range of (0, 1). Plots were generated in GraphPad Prism.

### 4.9. Tissue Culture

Cell culture well inserts were designed in SOLIDWORKS as a 6.25 mm diameter, 1 mm tall disc and directly printed onto the build platform for easy removal and assembly into well plates. After removing from the build platform, parts were rinsed in IPA to remove excess resin. Well inserts were printed with lv-PDMS and lv-PDMS (no absorber) (19.2 w/w% 083, 80 w/w % DMS-R22, and 0.8 w/w% TPO-L) resins.

BioAssay Systems (Hayward, CA, USA) provided Corning black, clear-bottom tissue culture 96-well plates (catalog number 3603) (Corning, Inc., Corning, NY, USA). To assemble the 96-well plates, a 10:1 polymer to crosslinker mixture of Sylgard 184 was prepared and 50 mg was added to each well, which was baked for 2 h at 85 °C. The 3D-printed inserts were glued to the bottom of the wells to prevent lifting or movement in the wells during the cell viability studies. Inserts were glued by applying a small drop of lv-PDMS (no absorber) to the bottom of the well. Inserts were transferred with tweezers into the bottom of the well and then exposed to 385 nm UV light from the Asiga printer at full power (35 mW/cm^2^) for 5 min to secure the inserts. Some inserts appeared to have a bubble under the insert because of trapped air under the polymer. Wells were washed twice with IPA for 3 min to remove debris or uncured resin that was pushed up onto the walls of the well when adding the inserts. No-EtOH-extraction inserts were left alone, and EtOH -extraction inserts and Sylgard 184 were soaked in ethanol for 72 h total, exchanging the ethanol every 24 h with fresh solvent to extract unreacted groups and photoabsorbing compounds. The assembled 96-well plates were postcured in an Asiga Flash-type DR-301C UV exposure chamber for 30 min. EtOH-extraction inserts were postcured following extraction. The assembled plate layout can be found in Figure S5.

Prepared 96-well plates with Sylgard 194, and 3D-printed inserts were sent to a third-party company, BioAssay Systems, for in vitro tissue culture studies with HepG2 cells to determine cell proliferation and viability. The methods for HepG2 cell viability and CQBL-05K assay were provided from BioAssay Systems as follows: The fresh HepG2 cells obtained from ATCC were grown in T75 culture flasks with ATCC-recommended media supplemented with antibiotics (10 *v/v* % FBS, 1 *v/v*% streptomycin/penicillin, EMEM media, 5% CO_2_, and 37 °C) to <70% confluency. The Corning black, clear-bottom tissue culture 96-well plates were sent to the University of Michigan for the insertion of various growth scaffolds. The 96-well plates were returned to BioAssay Systems. Prior to use, the plates were microwave sterilized for 3 min on high with a heat sink [36]. Prior to seeding, all the wells were treated with 100 μL of a 50 μg/mL solution of fibronectin for one hour at room temperature. Following fibronectin treatment, the wells were rinsed three times with 100 μL of growth media, then immediately seeded with 10,000 cells per well. The plates were incubated for 24, 48, or 72 h as outlined above. Each time point (24, 48, and 72 h) and treatment was assayed in triplicate with the CQBL-05K assay. Images of the well plates at 0 (immediately after seeding), 48, and 72 h are shown in Figure S6.

For the CQBL assay, the wells were treated with CellQuanti-BlueTM reagent for 1 h, and then 50 μL of the liquid from each well was transferred to a fresh fluorescence plate and scanned at 530 nm excitation/590 nm emission and 570 nm cutoff filter on a Molecular Devices SpectraMax M2.

The plot for each treatment minus background was analyzed and generated using GraphPad Prism (*n* = 3). Two-way ANOVA (α < 0.05) with Tukey’s multiple comparisons test was used to determine significance. A table summarizing the results of this analysis (Table S2) and the multiple comparisons (Table S3) can be found in the Supplementary Materials.

### 4.10. Microfluidic Prints

CAD for the biomimetic, branching device, and droplet generator device was performed in SOLIDWORKS (Figures S7 and S8 contain dimensioned drawings of the parts). Parts were printed with the lv-PDMS resin onto silanized glass slides with an Asiga MAX 27X UV printer. Parts were postprocessed by washing in IPA to remove excess resin. Parts were then assembled with 0.062 in ID and 0.125 in OD silastic tubing (LIVEO™ Silicone Laboratory Tubing, Avantor, Radnor, PA, USA) and 3140 RTV Silicone Conformal Coating glue (Ellsworth Adhesives, Germantown, WI, USA). After the glue dried, parts were flushed with IPA to remove excess resin from the internal channels. To encourage clearing of microchannels, parts could be placed on a continuous IPA circuit with a peristaltic roller pump (Masterflex 7523-80 Peristaltic Pump, Cole Parmer, Vernon Hills, IL, USA). Once the device was cleared of uncured resin, the parts were filled by flowing food dye in deionized water through the part for visualization, and images were taken on an iPhone 13 mini and/or Dino-Lite digital microscope.

## 5. Conclusions

The lv-PDMS resin presented in this paper promises streamlined and automated fabrication of microfluidic devices via 3D printing. The high resolution of this material combined with its other attractive properties, such as elasticity, gas permeability, optical clarity, and biocompatibility, seek to replace the laborious traditional microfabrication techniques with Sylgard 184. The intersection of 3D printing and microfluidics is soon to be realized as a universal microfabrication approach as higher-resolution materials, such as lv-PDMS, become available.

## 6. Patents

J. Potkay, E. Fleck. Photocurable resin for high-resolution 3-d printing, Application #17/332,655, Inventor, Filed: 05/27/2021.

## Figures and Tables

**Table 1:** Mechanical properties of lv-PDMS, hv-PDMS, and Sylgard 184.

Resin	Elongation at Break (%)	Young’s Modulus (MPa)
Sylgard 184	135.2 ± 27.27	0.50 ± 0.10
hv-PDMS	12.04 ± 1.72	11.48 ± 0.11
lv-PDMS	58.62 ± 18.80	0.30 ± 0.04

**Table 2:** Comparison of print resolution and material properties of DLP 3D-printing resins with Sylgard 184.

	Resin Material	Penetration Depth (μm)	Membrane Thickness (μm)	Channel Height (μm)	Young’s Modulus (MPa)	Elongation at Break (%)	O_2_/CO_2_ Permeability (Barrers)	Optical Transmission ^a^ (%)
This work (2023)	PDMS	47	31	39	0.3	58	596/3071	>80
Sylgard 184	PDMS	NA	NA	NA	1.3–3.0 [21]	140 [21]	586/2270 [28]	>90
Fleck (2021) [19]	PDMS	23	20	60	11.5	12	--	>75
Gonzalez (2020) [23]	PDMS	--	--	400	1.4	63	--	--
Bhattacharjee (2018) [22]	PDMS	546 *	330	2000	0.5	40–160	#	--
Femmer (2014) [29]	PDMS	293	840	>1000	11.5	--	523/2611	--
Kuo (2019) [16]	PEGDA	15	20	500	130 **	--	##	>80
Gong (2017) [15]	PEGDA	8	--	18	7	--	##	--

^a^: Reported for 400–700 nm wavelengths; --: not reported; NA: not applicable; *: not reported, calculated from data presented in Figure 2B from Bhattacharjee, N., et al.; #: demonstrated O_2_ permeability comparable to Sylgard 184 via fluorescence quenching with an oxygen sensitive fluorophore; ##: PEGDA O_2_ and CO_2_ permeability is not reported but varied with molecular weight and can range from 1 to 10 and 1 to 100 Barrers, respectively [30]; **: Young’s modulus is reported for PEG-DA-258. Young’s modulus for PEG-DA-258 resin with additives that was used for printing is not reported.

## Data Availability

Data will be provided upon request to the corresponding authors.

## Supplementary Materials

The following supporting information can be downloaded at: https://www.mdpi.com/article/10.3390/mi14040773/s1, Table S1: Rheology sample compositions; Figure S1: Dimensions of 3D printed microfluidic characterization print; Figure S2: Printing resolution characterization; Figure S3: Tensile testing set up; Figure S4: Dimensions of 3D printed tensile bars. Figure S5: Plate layout of 96-well plate with 3D printed inserts; Figure S6: HepG2 well plate photos; Figure S7: Dimensions of 3D printed biomimetic branching network; Figure S8: Dimensions of 3D printed droplet generator; Table S2: Two-way ANOVA results for HepG2 cell viability study; Table S3: Two-way ANOVA Tukey’s multiple comparisons results for HepG2 cell viability study; Video S1: Tensile bar elasticity. Video S2: Tensile testing.
