# Supplementary material for: Low-Viscosity Polydimethylsiloxane Resin for Facile 3D Printing of Elastomeric Microfluidics
Source: Micromachines (Basel). 2023 Mar 30;14(4):773. doi: 10.3390/mi14040773 (PMC10142493; doi:10.3390/mi14040773)
Supplement: Supplementary file 1 [file micromachines-14-00773-s001.zip › micromachines-2252200-supplementary Table S1.pdf]

Supplementary

Table S1 Rheology Sample Compositions

	DMS-R22 (w/w%)	RMS-083 (w/w%)	Sudan I (w/w%)	ITX (w/w%)	TPO-L (w/w%)
hv-PDMS	0	98.6	0.2	0.4	0.8
40 w/w% diluent	40	58.71	0.09	0.4	0.8
lv-PDMS	80	18.71	0.09	0.4	0.8
Diluent only	98.71	0	0.09	0.4	0.8

Figure S1 Dimensions of 3D printed microfluidic characterization print

Part was designed in SOLIDWORKS. All dimensions are in microns.

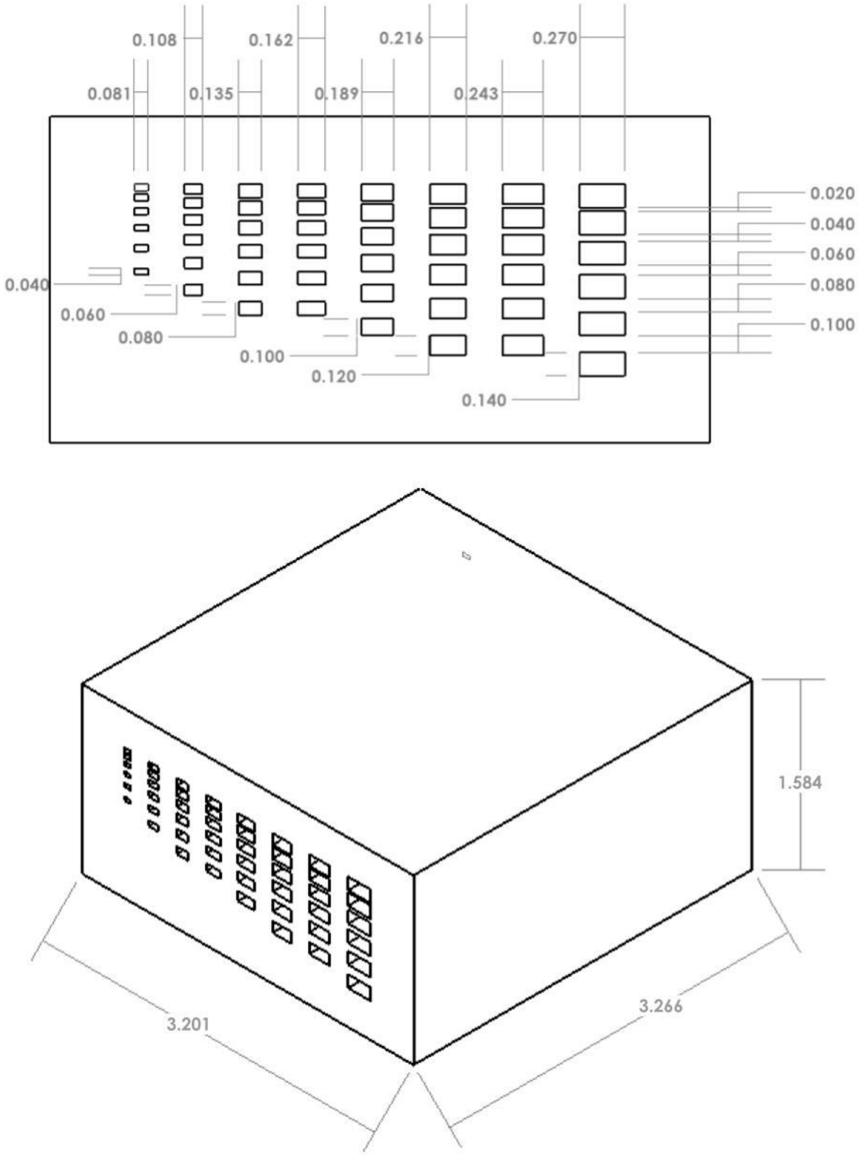

Figure S2 Printing Resolution Characterization

Dimensions of microfluidic characterization print (TOP) and XY pixel resolution print with constant spacing and varying pillar width (MIDDLE) and constant pillar width and varied spacing (BOTTOM).

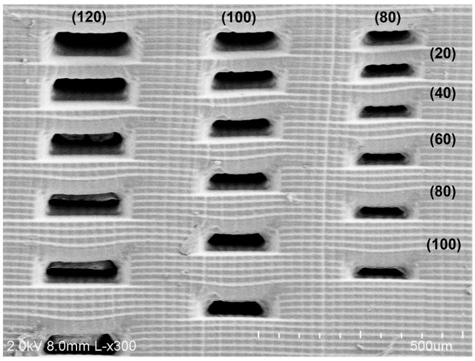

	Designed (µm)	Actual (µm)		
Channel Height	80	38.4	+/-	2.0
	100	54.3	+/-	2.5
	120	68.0	+/-	4.1
Membrane Thickness	20	30.9	+/-	0.3
	40	58.1	+/-	0.5
	60	77.7	+/-	1.0
	80	94.4	+/-	0.2
	100	113.1	+/-	0.5

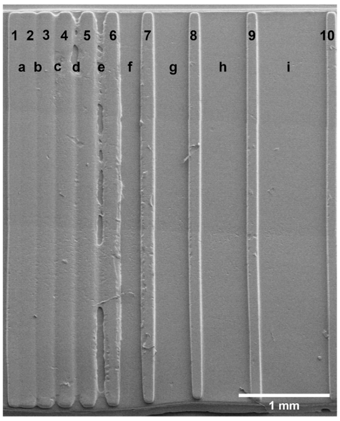

		1	2	3	4	5	6	7	8	9	10
Pillars	Actual (µm)	--	--	--	--	--	163.6 +/- 18.7	143.6 +/- 9.3	118.9 +/- 7.1	106.5 +/- 4.7	112.7 +/- 7.1
	Designed (pixels)	162 (6)	162 (6)	162 (6)	162 (6)	162 (6)	162 (6)	162 (6)	162 (6)	162 (6)	162 (6)
		a	b	c	d	e	f	g	h	i	
Spaces	Actual (µm)	--	--	--	--	--	226.9 +/- 16.0	393.6 +/- 16.7	510.8 +/- 9.6	759.3 +/- 4.7	
	Designed (pixels)	27 (1)	54 (2)	81 (3)	108 (4)	135 (5)	270 (10)	405 (15)	540 (20)	810 (30)	

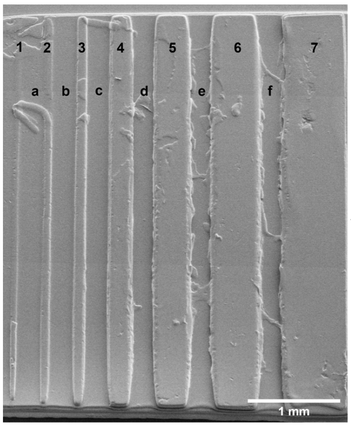

		1	2	3	4	5	6	7
Pillars	Actual (µm)	56.4 +/- 7.0	79.8 +/- 4.1	118.9 +/- 5.8	255.1 +/- 4.9	399.1 +/- 10.7	527.4 +/- 12.9	694.1 +/- 32.0
	Designed (pixels)	81 (3)	108 (4)	135 (5)	270 (10)	405 (15)	540 (20)	810 (30)
		a	b	c	d	e	f	
Spaces	Actual (µm)	266.8 +/- 8.2	258.1 +/- 4.2	244.1 +/- 4.7	226.9 +/- 6.8	237.1 +/- 8.5	247.4 +/- 15	
	Designed (pixels)	270 (10)	270 (10)	270 (10)	270 (10)	270 (10)	270 (10)	

Figure S3 Tensile testing setup

Tensile tests were performed using a TA×XT-PLUS Texture Analyser. The analyser has a load cell capacity of 30 kg, distance capacity from 0.1 - 295 mm, distance resolution of 0.001 mm, and speed capacity from 0.01 - 40 mm/s. Tests were performed in tension at a rate of 0.2 mm/s until fracture. The setup of a 3D printed tensile bar in tension is shown below and a short movie in 2x speed is available (Video S2).

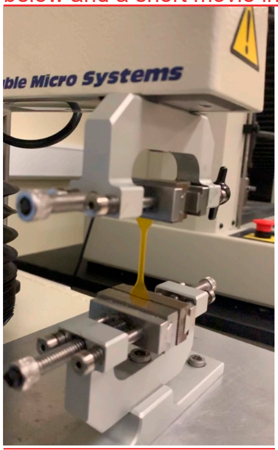

Figure S3 S4 Dimensions of 3D printed tensile bars

Part was designed in SOLIDWORKS. Dimensioned drawing of tensile bars printed for mechanical testing (left). Bars were drawn designed according to ASTM D412 die C and a 0.44x scalar was applied so that the bars would fit within the build area of the Asiga MX X27 UV printer. The acrylic mold used to fabricate Sylgard 184 samples is shown on the right.

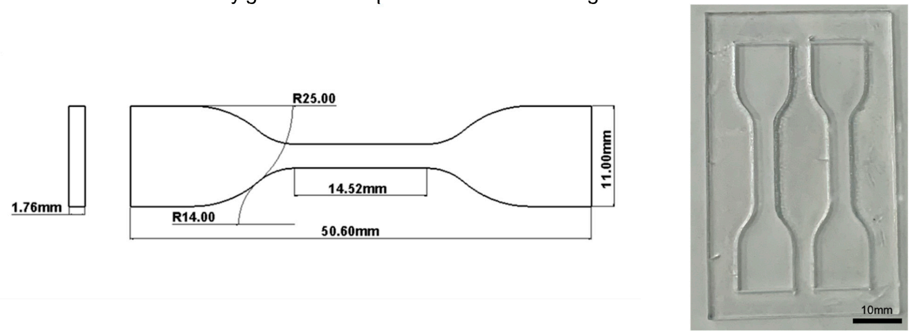

Formatted: Font: (Default) Arial, Underline

Formatted: Font: (Default) Arial

Formatted: Font: (Default) Arial

Formatted: Font: (Default) Arial

Formatted: Font: (Default) Arial

Figure S4 S5 Dimensions ofPlate layout of 96 well plate with 3D printed 3-well tissue culture platesinserts

Part was designed in SOLIDWORKS. All dimensions are in millimeters.

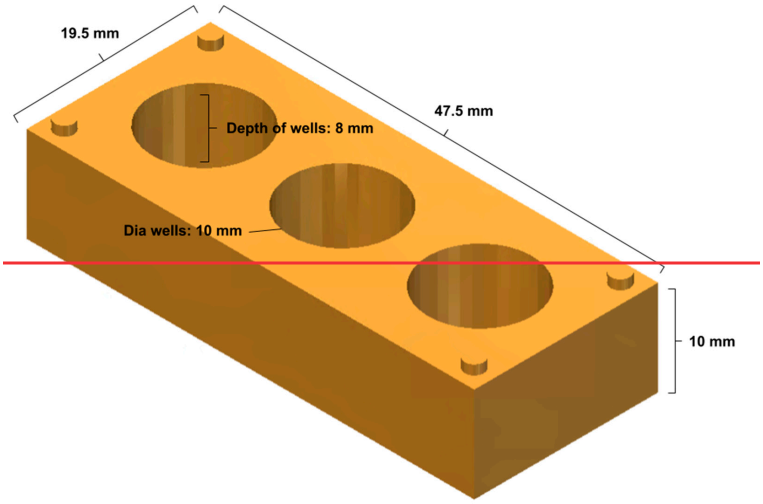

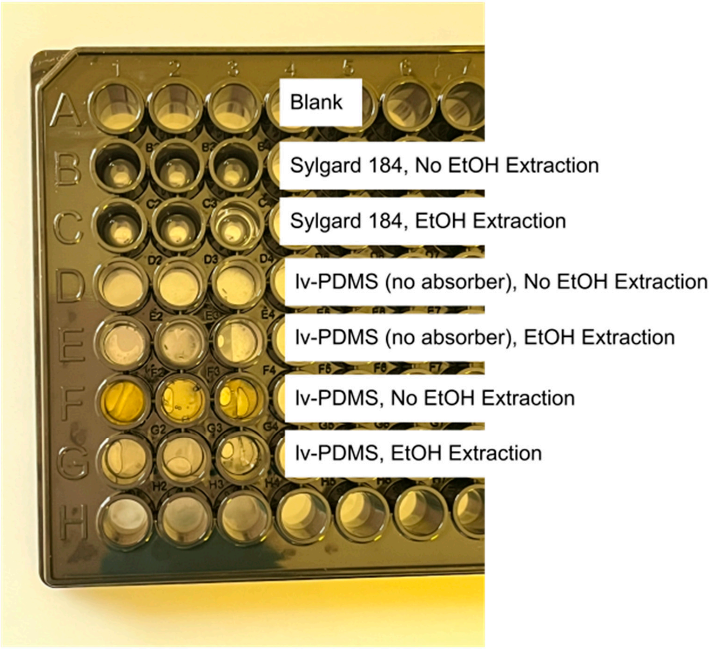

Formatted: Left

Figure ~~S5~~ S6 HepG2 well plate photos at 0 hours (top), 48 hours (middle), and 72 hours (bottom)

0 Hours

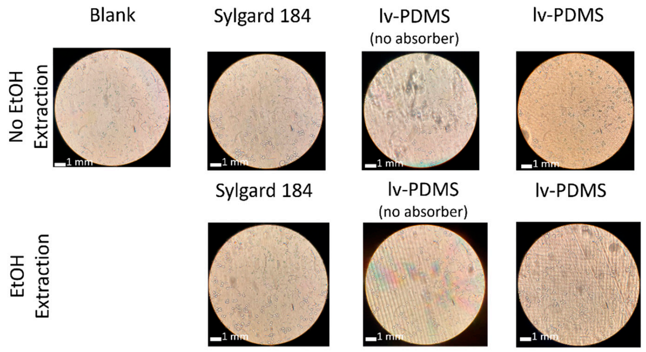

48 Hours

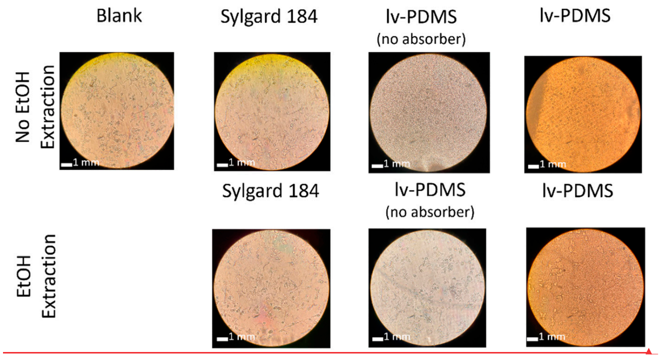

72 Hours

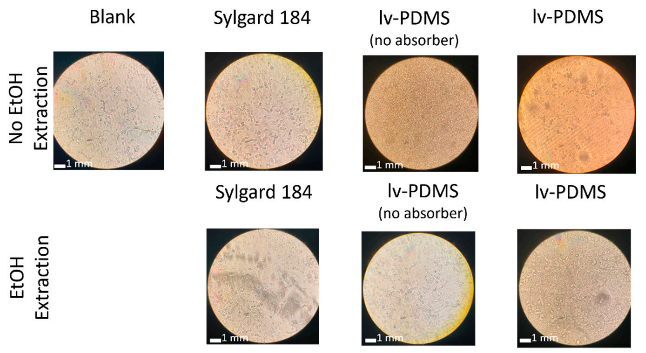

Formatted: Font: Italic

Formatted: No underline

Formatted: Font: Italic, No underline

Formatted: Font: Not Italic

Formatted: Font: Italic, No underline

Formatted: No underline

Figure S7 Dimensions of 3D printed biomimetic branching network

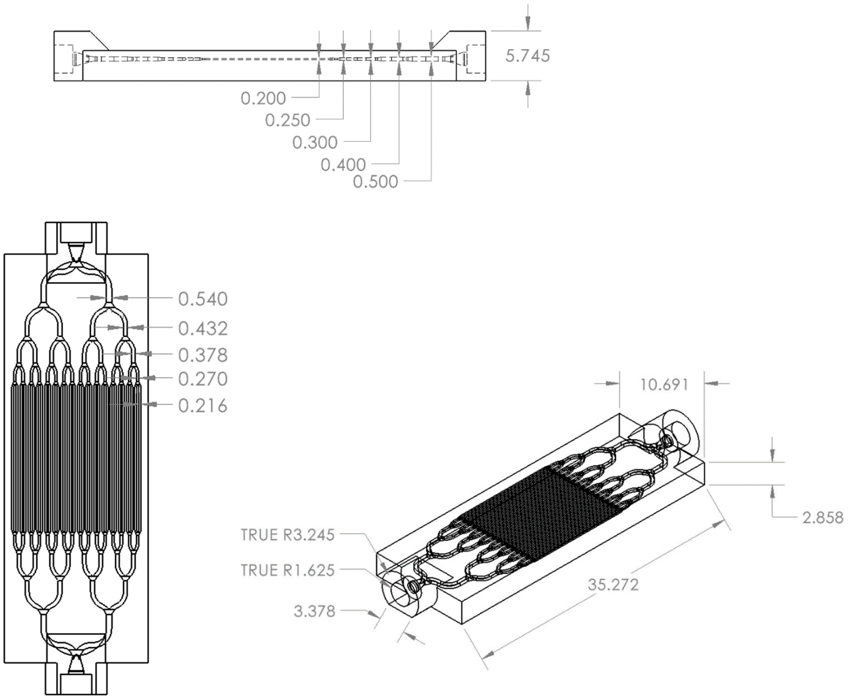

Formatted: Underline

Figure ~~S6~~ S8 Dimensions of 3D printed droplet generator

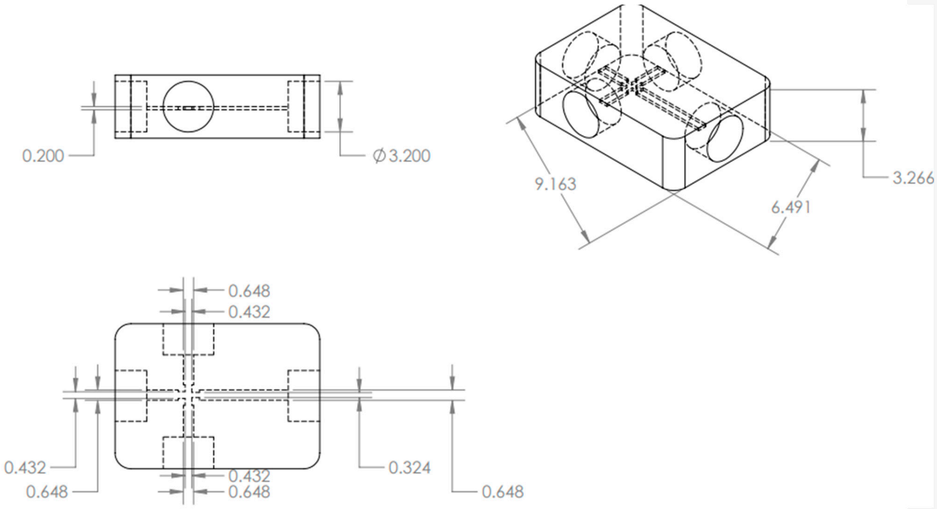

Table S2 2-way ANOVA results for HepG2 cell viability study

Table Analyzed	Baseline-corrected of HepG2 with fibronectin transfer RFU				
Two-way RM ANOVA	Matching: Stacked				
Assume sphericity?	No				
Alpha	0.05				
Source of Variation	% of total variation	P value	P value summary	Significant?	Geisser-Greenhouse's epsilon
Time x Well condition	32	<0.0001	****	Yes	
Time	36	<0.0001	****	Yes	0.80
Well condition	28	<0.0001	****	Yes	
Cell viability	1.1	0.6216	ns	No	
ANOVA table	SS	DF	MS	F (DFn, DFd)	P value
Time x Well condition	83246	12	6937	F (12, 28) = 29	P<0.0001
Time	95719	2	47860	F (1.6, 22) = 198	P<0.0001
Well condition	74309	6	12385	F (6, 14) = 61	P<0.0001
Cell viability	2846	14	203	F (14, 28) = 0.84	P=0.6216
Residual	6754	28	241		
Data summary					
Number of columns (Well condition)	7				
Number of rows (Time)	3				
Number of subjects (Cell viability)	21				
Number of missing values	0				

Formatted: Font: 9 pt
Formatted Table
Formatted: Font: 9 pt
Formatted: Font: 9 pt
Formatted Table
Formatted: Font: 9 pt
Formatted: Font: 9 pt
Formatted: Font: 9 pt
Formatted: Font: 9 pt
Formatted Table
Formatted: Font: 9 pt
Formatted: Font: 9 pt
Formatted: Font: 9 pt
Formatted: Font: 9 pt
Formatted: Font: 9 pt
Formatted: Font: 9 pt
Formatted Table
Formatted: Font: 9 pt
Formatted: Font: 9 pt
Formatted: Font: 9 pt
Formatted: Font: 9 pt
Formatted: Font: 9 pt
Formatted: Font: 9 pt
Formatted: Font: 9 pt
Formatted: Font: 9 pt
Formatted: Font: 9 pt
Formatted: Font: 9 pt
Formatted: Font: 9 pt
Formatted: Font: 9 pt
Formatted: Font: 9 pt
Formatted: Font: 9 pt

Table S3 2-way ANOVA Tukey's multiple comparisons results for HepG2 cell viability study

Within each row, compare columns (simple effects within rows)					
Number of families	3				
Number of comparisons per family	21				
Alpha	0.05				
Tukey's multiple comparisons test	Mean Diff.	95.00% CI of diff.	Below threshold?	Summary	Adjusted P Value
24					
Blank vs. Sylgard 184, - EtOH	10	-15 to 35	No	ns	0.3495
Blank vs. Sylgard 184, + EtOH	14	-11 to 39	No	ns	0.2521
Blank vs. lv-PDMS (no absorber), - EtOH	5.1	-26 to 36	No	ns	0.9576
Blank vs. lv-PDMS (no absorber), + EtOH	14	-12 to 40	No	ns	0.2976
Blank vs. lv-PDMS, - EtOH	-56	-79 to -34	Yes	**	0.0019
Blank vs. lv-PDMS, + EtOH	-19	-51 to 12	No	ns	0.1958
Sylgard 184, - EtOH vs. Sylgard 184, + EtOH	4.3	-22 to 31	No	ns	0.8850
Sylgard 184, - EtOH vs. lv-PDMS (no absorber), - EtOH	-4.9	-42 to 32	No	ns	0.9236
Sylgard 184, - EtOH vs. lv-PDMS (no absorber), + EtOH	3.8	-25 to 32	No	ns	0.9373
Sylgard 184, - EtOH vs. lv-PDMS, - EtOH	-66	-86 to -46	Yes	**	0.0022
Sylgard 184, - EtOH vs. lv-PDMS, + EtOH	-29	-68 to 8.8	No	ns	0.0873
Sylgard 184, + EtOH vs. lv-PDMS (no absorber), - EtOH	-9.1	-40 to 22	No	ns	0.7249
Sylgard 184, + EtOH vs. lv-PDMS (no absorber), + EtOH	-0.49	-27 to 26	No	ns	>0.9999
Sylgard 184, + EtOH vs. lv-PDMS, - EtOH	-70	-94 to -47	Yes	***	0.0010
Sylgard 184, + EtOH vs. lv-PDMS, + EtOH	-34	-65 to -2.1	Yes	*	0.0407
lv-PDMS (no absorber), - EtOH vs. lv-PDMS (no absorber), + EtOH	8.7	-23 to 40	No	ns	0.7764
lv-PDMS (no absorber), - EtOH vs. lv-PDMS, - EtOH	-61	-93 to -30	Yes	**	0.0062
lv-PDMS (no absorber), - EtOH vs. lv-PDMS, + EtOH	-25	-59 to 9.3	No	ns	0.1361
lv-PDMS (no absorber), + EtOH vs. lv-PDMS, - EtOH	-70	-95 to -45	Yes	**	0.0014
lv-PDMS (no absorber), + EtOH vs. lv-PDMS, + EtOH	-33	-65 to -1.4	Yes	*	0.0434
lv-PDMS, - EtOH vs. lv-PDMS, + EtOH	37	4.7 to 69	Yes	*	0.0336
48					
Blank vs. Sylgard 184, - EtOH	-14	-60 to 31	No	ns	0.4552
Blank vs. Sylgard 184, + EtOH	-12	-82 to 58	No	ns	0.9285
Blank vs. lv-PDMS (no absorber), - EtOH	30	-7.3 to 68	No	ns	0.0956
Blank vs. lv-PDMS (no absorber), + EtOH	-4.2	-53 to 45	No	ns	0.9985
Blank vs. lv-PDMS, - EtOH	36	-26 to 99	No	ns	0.2146
Blank vs. lv-PDMS, + EtOH	-71	-140 to -2.5	Yes	*	0.0452
Sylgard 184, - EtOH vs. Sylgard 184, + EtOH	2.3	-86 to 91	No	ns	>0.9999
Sylgard 184, - EtOH vs. lv-PDMS (no absorber), - EtOH	45	14 to 75	Yes	*	0.0197
Sylgard 184, - EtOH vs. lv-PDMS (no absorber), + EtOH	10	-51 to 71	No	ns	0.8191

Formatted Table

Sylgard 184, - EtOH vs. lv-PDMS, - EtOH	50	-30 to 130	No	ns	0.1250
Sylgard 184, - EtOH vs. lv-PDMS, + EtOH	-57	-144 to 30	No	ns	0.1154
Sylgard 184, + EtOH vs. lv-PDMS (no absorber), - EtOH	42	-33 to 118	No	ns	0.1882
Sylgard 184, + EtOH vs. lv-PDMS (no absorber), + EtOH	7.6	-60 to 76	No	ns	0.9929
Sylgard 184, + EtOH vs. lv-PDMS, - EtOH	48	-23 to 119	No	ns	0.1657
Sylgard 184, + EtOH vs. lv-PDMS, + EtOH	-59	-133 to 14	No	ns	0.0990
lv-PDMS (no absorber), - EtOH vs. lv-PDMS (no absorber), + EtOH	-35	-84 to 15	No	ns	0.1316
lv-PDMS (no absorber), - EtOH vs. lv-PDMS, - EtOH	5.7	-61 to 73	No	ns	0.9943
lv-PDMS (no absorber), - EtOH vs. lv-PDMS, + EtOH	-102	-176 to -27	Yes	*	0.0229
lv-PDMS (no absorber), + EtOH vs. lv-PDMS, - EtOH	40	-22 to 103	No	ns	0.1805
lv-PDMS (no absorber), + EtOH vs. lv-PDMS, + EtOH	-67	-134 to 0.17	No	ns	0.0504
lv-PDMS, - EtOH vs. lv-PDMS, + EtOH	-107	-178 to -37	Yes	*	0.0114
72					
Blank vs. Sylgard 184, - EtOH	-101	-182 to -21	Yes	*	0.0262
Blank vs. Sylgard 184, + EtOH	-82	-161 to -2.7	Yes	*	0.0448
Blank vs. lv-PDMS (no absorber), - EtOH	4.8	-108 to 118	No	ns	>0.9999
Blank vs. lv-PDMS (no absorber), + EtOH	-92	-171 to -14	Yes	*	0.0296
Blank vs. lv-PDMS, - EtOH	53	-28 to 133	No	ns	0.1515
Blank vs. lv-PDMS, + EtOH	-192	-322 to -62	Yes	*	0.0152
Sylgard 184, - EtOH vs. Sylgard 184, + EtOH	20	-43 to 82	No	ns	0.6593
Sylgard 184, - EtOH vs. lv-PDMS (no absorber), - EtOH	106	-21 to 233	No	ns	0.0770
Sylgard 184, - EtOH vs. lv-PDMS (no absorber), + EtOH	8.8	-46 to 63	No	ns	0.9664
Sylgard 184, - EtOH vs. lv-PDMS, - EtOH	154	108 to 200	Yes	***	0.0005
Sylgard 184, - EtOH vs. lv-PDMS, + EtOH	-91	-240 to 58	No	ns	0.1481
Sylgard 184, + EtOH vs. lv-PDMS (no absorber), - EtOH	86	-30 to 203	No	ns	0.1103
Sylgard 184, + EtOH vs. lv-PDMS (no absorber), + EtOH	-11	-75 to 53	No	ns	0.9645
Sylgard 184, + EtOH vs. lv-PDMS, - EtOH	134	72 to 197	Yes	**	0.0041
Sylgard 184, + EtOH vs. lv-PDMS, + EtOH	-110	-246 to 25	No	ns	0.0848
lv-PDMS (no absorber), - EtOH vs. lv-PDMS (no absorber), + EtOH	-97	-217 to 23	No	ns	0.0855
lv-PDMS (no absorber), - EtOH vs. lv-PDMS, - EtOH	48	-79 to 175	No	ns	0.3853
lv-PDMS (no absorber), - EtOH vs. lv-PDMS, + EtOH	-197	-329 to -64	Yes	*	0.0126
lv-PDMS (no absorber), + EtOH vs. lv-PDMS, - EtOH	145	91 to 199	Yes	**	0.0016
lv-PDMS (no absorber), + EtOH vs. lv-PDMS, + EtOH	-100	-240 to 41	No	ns	0.1140
lv-PDMS, - EtOH vs. lv-PDMS, + EtOH	-245	-394 to -96	Yes	*	0.0156
Test details	Mean 1	Mean 2	Mean Diff.	SE of diff.	N1
24					

Blank vs. Sylgard 184, - EtOH	78	68	10	3.7	3
Blank vs. Sylgard 184, + EtOH	78	64	14	5.0	3
Blank vs. lv-PDMS (no absorber), - EtOH	78	73	5.1	5.9	3
Blank vs. lv-PDMS (no absorber), + EtOH	78	64	14	5.1	3
Blank vs. lv-PDMS, - EtOH	78	134	-56	4.5	3
Blank vs. lv-PDMS, + EtOH	78	98	-19	6.0	3
Sylgard 184, - EtOH vs. Sylgard 184, + EtOH	68	64	4.3	3.8	3
Sylgard 184, - EtOH vs. lv-PDMS (no absorber), - EtOH	68	73	-4.9	4.9	3
Sylgard 184, - EtOH vs. lv-PDMS (no absorber), + EtOH	68	64	3.8	4.1	3
Sylgard 184, - EtOH vs. lv-PDMS, - EtOH	68	134	-66	3.2	3
Sylgard 184, - EtOH vs. lv-PDMS, + EtOH	68	98	-29	5.0	3
Sylgard 184, + EtOH vs. lv-PDMS (no absorber), - EtOH	64	73	-9.1	6.0	3
Sylgard 184, + EtOH vs. lv-PDMS (no absorber), + EtOH	64	64	-0.49	5.3	3
Sylgard 184, + EtOH vs. lv-PDMS, - EtOH	64	134	-70	4.6	3
Sylgard 184, + EtOH vs. lv-PDMS, + EtOH	64	98	-34	6.1	3
lv-PDMS (no absorber), - EtOH vs. lv-PDMS (no absorber), + EtOH	73	64	8.7	6.1	3
lv-PDMS (no absorber), - EtOH vs. lv-PDMS, - EtOH	73	134	-61	5.6	3
lv-PDMS (no absorber), - EtOH vs. lv-PDMS, + EtOH	73	98	-25	6.8	3
lv-PDMS (no absorber), + EtOH vs. lv-PDMS, - EtOH	64	134	-70	4.8	3
lv-PDMS (no absorber), + EtOH vs. lv-PDMS, + EtOH	64	98	-33	6.2	3
lv-PDMS, - EtOH vs. lv-PDMS, + EtOH	134	98	37	5.7	3
48					
Blank vs. Sylgard 184, - EtOH	113	127	-14	6.0	3
Blank vs. Sylgard 184, + EtOH	113	125	-12	12	3
Blank vs. lv-PDMS (no absorber), - EtOH	113	82	30	7.1	3
Blank vs. lv-PDMS (no absorber), + EtOH	113	117	-4.2	9.5	3
Blank vs. lv-PDMS, - EtOH	113	77	36	11	3
Blank vs. lv-PDMS, + EtOH	113	184	-71	12	3
Sylgard 184, - EtOH vs. Sylgard 184, + EtOH	127	125	2.3	11	3
Sylgard 184, - EtOH vs. lv-PDMS (no absorber), - EtOH	127	82	45	4.5	3
Sylgard 184, - EtOH vs. lv-PDMS (no absorber), + EtOH	127	117	10	7.6	3
Sylgard 184, - EtOH vs. lv-PDMS, - EtOH	127	77	50	9.7	3
Sylgard 184, - EtOH vs. lv-PDMS, + EtOH	127	184	-57	10	3
Sylgard 184, + EtOH vs. lv-PDMS (no absorber), - EtOH	125	82	42	11	3
Sylgard 184, + EtOH vs. lv-PDMS (no absorber), + EtOH	125	117	7.6	13	3
Sylgard 184, + EtOH vs. lv-PDMS, - EtOH	125	77	48	14	3
Sylgard 184, + EtOH vs. lv-PDMS, + EtOH	125	184	-59	15	3
lv-PDMS (no absorber), - EtOH vs. lv-PDMS (no absorber), + EtOH	82	117	-35	8.6	3

lv-PDMS (no absorber), - EtOH vs. lv-PDMS, - EtOH	82	77	5.7	10	3
lv-PDMS (no absorber), - EtOH vs. lv-PDMS, + EtOH	82	184	-102	11	3
lv-PDMS (no absorber), + EtOH vs. lv-PDMS, - EtOH	117	77	40	12	3
lv-PDMS (no absorber), + EtOH vs. lv-PDMS, + EtOH	117	184	-67	13	3
lv-PDMS, - EtOH vs. lv-PDMS, + EtOH	77	184	-107	14	3
72					
Blank vs. Sylgard 184, - EtOH	119	220	-101	14	3
Blank vs. Sylgard 184, + EtOH	119	200	-82	15	3
Blank vs. lv-PDMS (no absorber), - EtOH	119	114	4.8	21	3
Blank vs. lv-PDMS (no absorber), + EtOH	119	211	-92	15	3
Blank vs. lv-PDMS, - EtOH	119	66	53	14	3
Blank vs. lv-PDMS, + EtOH	119	311	-192	23	3
Sylgard 184, - EtOH vs. Sylgard 184, + EtOH	220	200	20	12	3
Sylgard 184, - EtOH vs. lv-PDMS (no absorber), - EtOH	220	114	106	19	3
Sylgard 184, - EtOH vs. lv-PDMS (no absorber), + EtOH	220	211	8.8	11	3
Sylgard 184, - EtOH vs. lv-PDMS, - EtOH	220	66	154	9.2	3
Sylgard 184, - EtOH vs. lv-PDMS, + EtOH	220	311	-91	21	3
Sylgard 184, + EtOH vs. lv-PDMS (no absorber), - EtOH	200	114	86	20	3
Sylgard 184, + EtOH vs. lv-PDMS (no absorber), + EtOH	200	211	-11	13	3
Sylgard 184, + EtOH vs. lv-PDMS, - EtOH	200	66	134	12	3
Sylgard 184, + EtOH vs. lv-PDMS, + EtOH	200	311	-110	22	3
lv-PDMS (no absorber), - EtOH vs. lv-PDMS (no absorber), + EtOH	114	211	-97	19	3
lv-PDMS (no absorber), - EtOH vs. lv-PDMS, - EtOH	114	66	48	19	3
lv-PDMS (no absorber), - EtOH vs. lv-PDMS, + EtOH	114	311	-197	26	3
lv-PDMS (no absorber), + EtOH vs. lv-PDMS, - EtOH	211	66	145	11	3
lv-PDMS (no absorber), + EtOH vs. lv-PDMS, + EtOH	211	311	-100	21	3
lv-PDMS, - EtOH vs. lv-PDMS, + EtOH	66	311	-245	21	3

Supplementary Videos

Video S1. Stretching of a tensile bar demonstrating elasticity of the material.

Video S2. Mechanical testing of a tensile bar at 2x speed.

Formatted: Underline

Formatted: No underline

Formatted: No underline

Formatted: No underline

Formatted: No underline

Formatted: Underline
